# Lamin microaggregates lead to altered mechanotransmission in progerin-expressing cells

**DOI:** 10.1080/19491034.2020.1802906

**Published:** 2020-08-20

**Authors:** Brooke E. Danielsson, Katie V. Tieu, Kranthidhar Bathula, Travis J. Armiger, Pragna S. Vellala, Rebecca E. Taylor, Kris Noel Dahl, Daniel E. Conway

**Affiliations:** a Department of Biomedical Engineering, Virginia Commonwealth University, Richmond, VA, USA; b Chemical Engineering, Carnegie Mellon University, Pittsburgh, PA, USA; c Department of Biomedical Engineering, Carnegie Mellon University, Pittsburgh, PA , USA; d Department of Mechanical Engineering, Carnegie Mellon University, Pittsburgh, PA, USA

**Keywords:** Nucleoskeleton mechanics, Hutchinson-Gilford progeria syndrome, nuclear structure

## Abstract

The nuclear lamina is a meshwork of intermediate filament proteins, and lamin A is the primary mechanical protein. An altered splicing of lamin A, known as progerin, causes the disease Hutchinson-Gilford progeria syndrome. Progerin-expressing cells have altered nuclear shapes and stiffened nuclear lamina with microaggregates of progerin. Here, progerin microaggregate inclusions in the lamina are shown to lead to cellular and multicellular dysfunction. We show with Comsol simulations that stiffened inclusions causes redistribution of normally homogeneous forces, and this redistribution is dependent on the stiffness difference and relatively independent of inclusion size. We also show mechanotransmission changes associated with progerin expression in cells under confinement and cells under external forces. Endothelial cells expressing progerin do not align properly with patterning. Fibroblasts expressing progerin do not align properly to applied cyclic force. Combined, these studies show that altered nuclear lamina mechanics and microstructure impacts cytoskeletal force transmission through the cell.

## Introduction

Increasingly detailed structural analysis of the nuclear lamina has emerged showing filament size [1,2], disparate network localization with the nuclear membrane [3] and nuclear pore association [4]. Mutations of *LMNA*, which codes for A-type lamins, cause numerous diseases impacting different tissue types depending on the mutation. However, the increasingly accurate information on lamin architecture is not necessarily coupled with a better understanding of how altered lamina structure relates to cellular and tissue level dysfunction. This disconnect between molecular assemblies and cellular dysfunction prevents a full characterization of disease pathologies and movement toward treatment and therapy options.

Hutchinson-Gilford progeria syndrome (HGPS) is a rare premature aging disease in children [5] caused by an autosomal dominant mutation in the *LMNA* gene. The mutation results in an alternate splicing of exon 11 leading to a loss of 50 amino acids in the tail domain [6]. This altered splicing occurs at extremely low but perceivable levels in wildtype cells as well, leading to a similar cellular phenotype in aged cells [7–9]. This splice variant of *LMNA* associated with HGPS is called progerin or Δ50 lamin A and retains a C-terminal farnesylation and carboxymethylation that mature lamin A loses during processing [10]. Ultimately, progerin expression leads to an accumulation of structural proteins in the lamina (progerin plus the retention of other lamins), altered nuclear shape, redistribution of heterochromatin, modified nuclear pore structure, alterations in gene expression and nuclear structural instability [7–9,11-13].

Important to this study, progerin expression changes both nuclear lamina mechanics and nuclear shape. Progerin-expressing cells have altered nuclear morphologies that have been described as blebs, wrinkles or folds [8,9,14,15]. Progerin expression and HGPS are associated with increased lamina stiffness [7–9]. It is unclear how an increase in a structural protein and a stiffening of the lamina could lead to the blebbed nuclear lamina, which is seemingly related to lamina fragility. Our studies and others have observed the formation of microaggregates of progerin within the lamina [16–18]. The goal of this study is to show the mechanical impacts of stiffened inclusions of progerin microaggregates and how these ultimately manifest in cells as mechanical dysfunction of the nuclear lamina. We aim to link the overaccumulation of progerin associated with HGPS to ultrastructure changes in the nuclear lamina and dysfunction in cells under force. Thus, changes in lamina structure could explain cellular and tissue level disease. We investigate strains in cells under confinement and cells under external forces. In considering our data and models correlating the formation of microaggregates of progerin to altered force propagation through the nucleus. We also show that cells expressing progerin do not align properly to external patterning or force cues, suggesting altered nuclear microstructure may impact cytoskeletal force transmission through the cell. These combined structural effects may have important functional consequences in HGPS and highlight the benefit of applying physical models to study biological systems to determine aspects of disease states.

## Materials and Methods

### Cell Culture and Transfection

For HUVEC studies, primary HUVEC (pooled, passages 3–5, Lonza, Basel, Switzerland) were grown in EGM-2 medium (Lonza, Basel, Switzerland). To express progerin in HUVEC an adenovirus was developed to express HA-tagged progerin (HA-progerin was a gift of Bryce Paschal [19]; adenovirus was prepared by Vector Biolabs, Malvern PA). The lowest level of adenovirus that infected nearly 100% of cells was used. To overexpress wild-type lamin A in HUVEC, lamin A adenovirus (based on RefSeq BC014507) was purchased from Vector Biolabs and used at an identical titer level as progerin. Western blots of lamin and progerin levels in HUVEC are shown in Supplemental Figure 1. For actin depolymerization studies, latrunculin A (Tocris, Bristol, United Kingdom) was added at 10 μM for reported times before cell fixation and labeling.

For fibroblasts studies, primary human dermal fibroblasts were cultured under 5% CO_2_ in DMEM (Thermofisher) supplemented with 15% FBS (Thermofisher). The primary fibroblast cell lines used in our studies included AG06299 (normal) and AG11513 F (HGPS patient with mutation in exon 11 of LMNA gene), obtained from NIA Aging Cell Repository, Coriell Institute.

### Micropatterning

HUVECs were seeded on micropatterned lines of width 20 or 40 µm, as previously described [20]. Briefly, the stamps used to micropattern fibronectin lines of 20 or 40 µm were made with polydimethylsiloxane (PDMS). Stamps were coated with fibronectin and were pressed onto a prepared coverslip. Once stamped, the coverslips were washed and treated with Pluronic F-127 to limit cell adhesion to only the fibronectin lines. Cells were then seeded onto the coverslip.

### Cell stretching

Fibroblasts cells were seeded onto UniFlex culture plates (FLEXCELL International Forporation, NC) coated with 60 ng/mL of Fibronectin (Sigma). The cells were exposed to uniaxial stretch, using the FlexCell 5000 (FLEXCELL International Corporation, NC) with 10% strain and frequency of 0.5 Hz for 24 hours.

### Cell Fixation, Immunocytochemistry Labeling and Western Blotting

Cells were fixed using 4% formaldehyde in phosphate buffered saline (PBS) and permeabilized using 0.2% Triton X-100 in PBS. For fluorescence microscopy experiments, cells were stained with 0.1 μg/mL Hoechst 33342 (ThermoFisher, Waltham, MA, USA) for DNA staining. HUVECs were stained with anti-lamin A/C antibody (cat # sc-7292, Santa Cruz Biotechnology, Dallas, TX, USA) for control cells or anti-HA antibody (cat # 901501, Biolegend, San Diego, CA, USA) for progerin-expressing cells with an Alexa Fluor 488 fluorescent secondary (cat # A-21202, ThermoFisher, Waltham, MA, USA). HUVECs were also stained with rhodamine phalloidin (cat # PHDR1, Cytoskeleton, Denver, CO, USA). The same antibodies were used for Western Blotting quantification of overexpression.

### Imaging and Analysis

Fibroblast cells were imaged using a Zeiss 710 LSM confocal at 20x and 63x and 1.4NA. Fixed HUVEC cells were imaged on a Nikon Eclipse TS100-F widefield fluorescence microscope with a 50x (1.4NA) oil immersion oil objective. Live HUVEC cells were imaged on a Leica DMI6000 inverted microscope using a 63x (1.4 NA) oil immersion objective. During imaging, the entire microscope environment was regulated by a Pecon live-cell imaging chamber heated to 37°C. Images were processed using ImageJ. Alignment was done for more than 100 cells per condition, multiple fields of view, random sampling per field of view using the angle tool. Manual angle analysis using the angle tool was preferred to avoid biasing for artificially bright actin stress fibers of other structures. Methodologies for wrinkle analysis are presented in Supplemental Figure 5 (for data in Figure 4). Again, 100 cells were considered but, in some cases, only 20% of cells had wrinkles, but some cells had numerous wrinkles.

### Simulations of inclusions

All modeling was completed in Comsol Multiphysics 5.3 using the two-dimensional (2D) plane stress module. The lamina was modeled as a uniform 2D elastic material with elastic modulus of 50 kPa. We chose this number based on Vaziri and Mofrad [21] with updates based on a new understanding of lamina thickness to be 10–100 nm based on super-resolution microscopy (from [2] and [3]); although scaling neglects the need for an absolute stiffness. Circular inclusions were modeled as linear elastic materials within the lamina. For this study, we approximated an infinite sheet by modeling a 4 μm by 4 μm square region of the membrane with a small inclusion ranging from 0.05 μm to 0.4 μm in diameter with varying stiffnesses. Unconfined 25% uniaxial strain in the x-direction (aside constraint holding the midline at y = 0) with Poisson ratio *ν* = 0.49 resulted in a stiffness profile around the y = 0 axis. Von Mises stresses are shown and peak midline stresses are reported.

## Results

### Progerin-expressing cells often show punctate inclusions or aggregates

**Figure 1.**
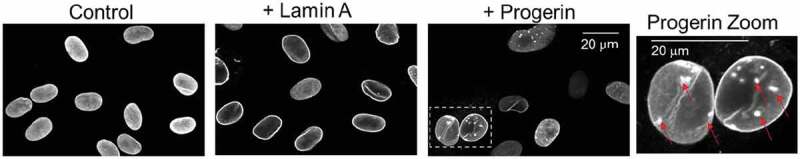
Punctate inclusions in progerin-expressing nuclei. Confocal images of HUVECs labeled via immunocytochemistry for endogenous LA/C, overexpressed LA and HA-tagged progerin. Control and LA cells show uniform equitorial labeling with come wrinkles due to actin fibers whereas progerin-expressing nuclei show punctate inclusions (arrows). The z-resolution for the lamin channel (488 nm) was chosen at 1.0 μm, so folds and puncta of the nuclear face may appear in the same confocal frame as the midline edge.

Similar to other studies [16–18], we consistently observe punctate inclusions of progerin in cells overexpressing progerin, which are not observed in lamin A overexpressing cells or control cells (Figure 1). Densitometry analysis of Western blots (Supplemental Figure 1) shows that lamin A overexpressing cells have 2.5x the lamin A compared with control cells; HA-progerin shows a 3x increase in A-type lamins with 2.5x of that increase from the HA-progerin. These aggregates at the nuclear lamina could be due to the altered stability of the tail domain of the progerin mutant compared to the wildtype [22], hydrophobic aggregation of the farnesyl tail of individual proteins within filaments, associations of the tail domains to specific regions of the inner nuclear membrane, or a combination of all of these [23]. We did not observe any large differences in actin organization with overexpression of progerin nor in patient cells (Supplemental Figure 2). These aggregates are observed (and likely exaggerated) in overexpressing cells, but regions of domain formation are also observed in patient cells (Supplemental Figure 3) [8].

### Simulations of stiffened inclusions show stress fields consistent with wrinkling

**Figure 2.**
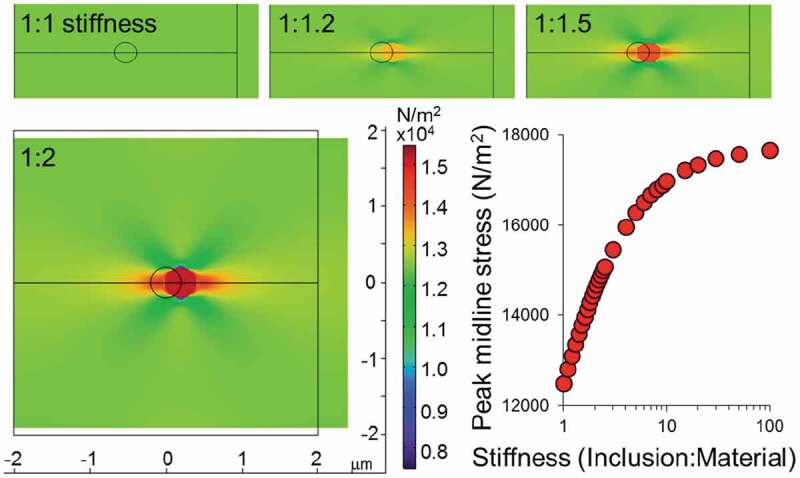
Strain on an inclusion of increased stiffness causes a line of increased stress normal to the imposed strain. Comsol simulation of a homogeneous structure with a stiffened inclusion is compressed in y and dilates in x. The resulting von Mises stiffness profile is shown for increasing inclusion stiffnesses (1, 1.2, 1.5 and 2x as stiff as the background material). The peak midline stress in the x-direction outside of the inclusion is plotted as a function of stiffness ratio.

To consider the consequence from aggregation of stiffening elements, we utilized a simulation to consider a stiffened region within the lamina, most simplistically modeled as a 2D continuum. We approximated the stiffness of the lamina (50 kPa, see Methods), added a stiffened inclusion within the uniform field (black circle), and then uniaxially strained in the x-direction and pinned along the black line at y = 0 (Figure 2). We then tracked the peak stress along the midline outside of the inclusion. High stresses in deviation from the bulk would lead to asymmetries that could initiate out of plane bending. Importantly, we found that the size of the inclusion (from 50 to 400 nm) did not influence the peak midline stress (Supplemental Figure 4). However, the ratio of stiffness of the inclusion to the material led to greater midline stresses, as expected (Figure 2). Thus, we suggest that the presence of stiffened inclusions leads to larger-scale stress features in the bulk of materials under strain. This continuum simulation shows the initiation of stresses, but it is important to state that any further analysis of instabilities or out-of-plane bending should be considered in a coursegrained filamentous model.

### Endothelial cells confined to one-dimensional patterns show differential lamina deformation

**Figure 3.**
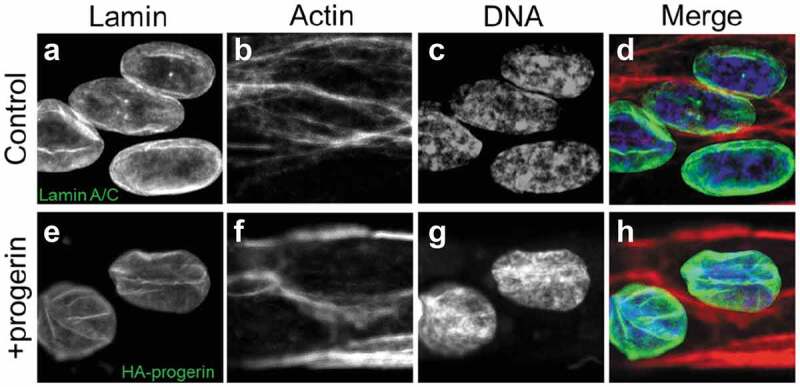
Confocal fluorescence microscopy confocal sections for cells patterned on lines. Fixed HUVECs were stained for Lamin (control) or HA (Progeria), and all cells were also stained for actin (phallodin) and DNA (Hoechst 33342). (a) Lamin A/C (control) stained with a lamin A/C antibody. (b) Control cell stained for actin to check the orientation of folds against the filament structures. (c) Lamin A control cells with Hoescht staining for DNA. (d) Merge of the lamin and actin channels shows nuclear alignment with the stripes and lamin folds coincident with the actin filaments. (e) Progerin-expressing cells stained with anti-HA to label HA-progerin express more wrinkles. (f) Progerin-expressing cells stained for actin to show the orientation of folds against the filament structures. (g) Progerin-expressing cells with Hoescht 33342 staining for DNA. (h) Merge of the lamin and actin shows lamin folds distinct from actin filaments. For both conditions the z-resolution for the lamin channel (488 nm) was chosen at 3.5 μm, actin channel (561 nm) 1.9 μm and DNA channel (405 nm) 1.3 μm.

To examine the role of extracellular perturbation on nuclear lamina reorganization, we considered how cells respond to growth on patterns. Endothelial cells were grown on patterned lines of 20 µm or 40 µm in order to ascertain the extent of deformation of the lamina network under cell confinement. Previously, patterning on lines of this thickness has been shown to exert forces on the nucleus from the cytoskeleton [20,24,25]. On 20 µm lines, nuclei are oblate and orient in the direction of the actin filaments. This orientation has been shown to be a direct function of the cellular confinement to patterning [24]. There are some folds in control lamina, but these coincide with actin filament structures (Figure 3(a–d)). Progerin-expressing cells show numerous folds and wrinkles in the nuclear lamina, but these dysmorphic structures do not align or co-register with confocal actin filament structures at a similar plane (Figure 3(e–h)).

**Figure 4.**
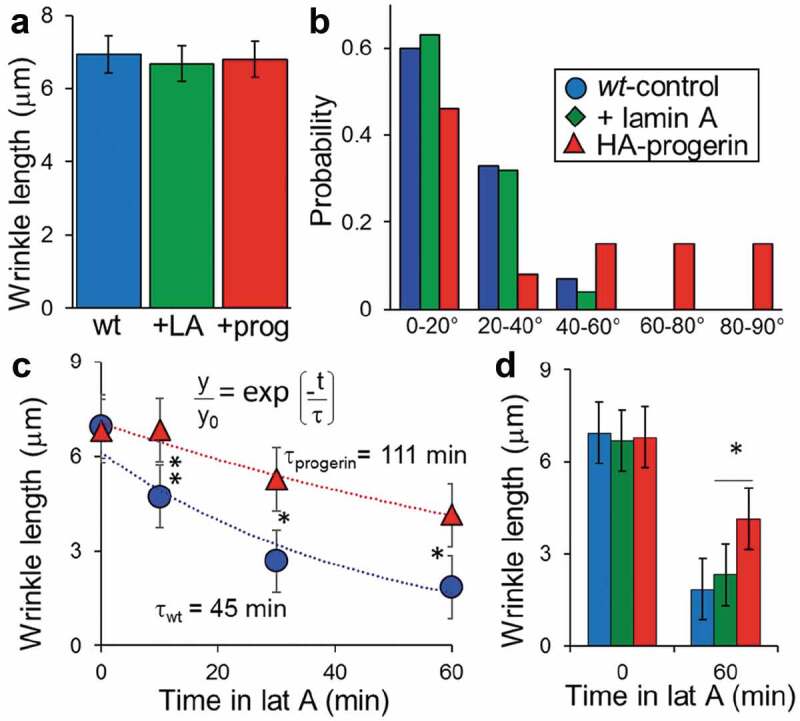
Formation of wrinkles for cells under one-dimensional confinement. (a) Length of deformations or wrinkles for control, exogenous lamin A or HA-progerin expressing endothelial cells cultured on 20 µm diameter stripes. (b) On 20 µm diameter stripes, wrinkles in control cells and exogenous lamin A expressing cells (+ lamin A) primarily align with the stripe axis whereas HA-progerin-expressing cells do not show preferred orientation. (c) On 20 µm diameter stripes, treatment with latrunculin A and fixation at different time points shows an exponential decay. (d) Fits of exponential decay shows the differential decay constants for control and exogenous lamin A versus HA-progerin cells. Fits same for 4 points as 2 points. 30–50 cells per condition considered. * indicates statistically similar *p* > 0.05; ** indicates 0.001 < *p* < 0.05; (c and d) no * indicates statistically different with *p* < 0.001 using unpaired Students t-test.

We quantified the dysmorphic structures or wrinkles observed in the lamina, visualized in Figure 3 along the length of the nucleus and compared them to the orientation of the nucleus (see schematic in Supplemental Figure 5A). Note that z-resolution of lamins was 3.5 μm – larger than normal confocal sections – to allow full visualization along the length of the nuclear lamina wrinkle. Earlier studies have suggested that cells under extreme loading conditions or, in this case, confinement may propagate wrinkle or fissure formations [25]. Lamin networks that are healthy have been found to deform uniformly under similar conditions [24]. For cells patterned on 20 µm stripes, wrinkles observed in the lamina (seen in Figure 3) were not statistically different for control and progerin-expressing endothelial cells (Figure 4(a)). As an additional control, we also overexpressed wild-type lamin A in cells to ensure that the results were from progerin expression and not from either increased lamin A or from viral treatment. Levels of exogenous lamin A, measured from confocal immunocytochemistry, were 204 ± 43% higher compared to wildtype cells. Endothelial cells grown on wider, 40 µm stripes without progerin did not show any wrinkles whereas progerin-expressing cells had wrinkles statistically similar to cells without progerin grown on 20 µm stripes (Supplemental Figure 6(a)).

In cells on 20 µm stripes, we also considered the orientation of the wrinkles (see Supplemental Figure 6(b)). Our data indicate that the most deformations in control nuclear lamina structures lie in the direction of the primary orientation of the cells with more than half at 0–20° (Figure 4(b)). This agrees with the organized actin cytoskeleton visible along the length of the stripes visible in the overlays (Figure 2). Conversely, progerin-expressing cells displayed angles ranging from 40° to 90° for many of these folds. For progerin-expressing cells on 40 µm stripes, there is an increased number of wrinkle formations in the range of 80–90°, which is nearly normal to the applied force from the actin cytoskeleton (Supplemental Figure 6(b)).

To compare control versus progerin-expressing cells, we considered cells on 20 µm stripes and quantified the wrinkles in the nuclei. In cells confined on the stripes, we depolymerized actin using latrunculin A, fixed cells at increasing time, and imaged the nuclear lamina in control and HA-progerin expressing cells. The actin depolymerized within a minute as expected but the wrinkles in nuclei took some time to be removed, likely based on the stiff mechanics of the nucleus. We plotted the length of wrinkles versus time after actin depolymerization treatment to determine if there was a difference in the loss of wrinkles. From the plot (Figure 4(c)), the wrinkle loss from both cases can be modeled as an exponential decay. Fits of exponential decay of control and HA-progerin are shown in Figure 4(c); progerin-expressing cells show a slower loss of wrinkles on a timescale of 111 min versus 45 min control cells. Exogenous-lamin A expressing cells are statistically similar to control at 0 and 60 min (Figure 4(d)).

### Fibroblasts fail to align when exposed to uniaxial stretch

**Figure 5.**
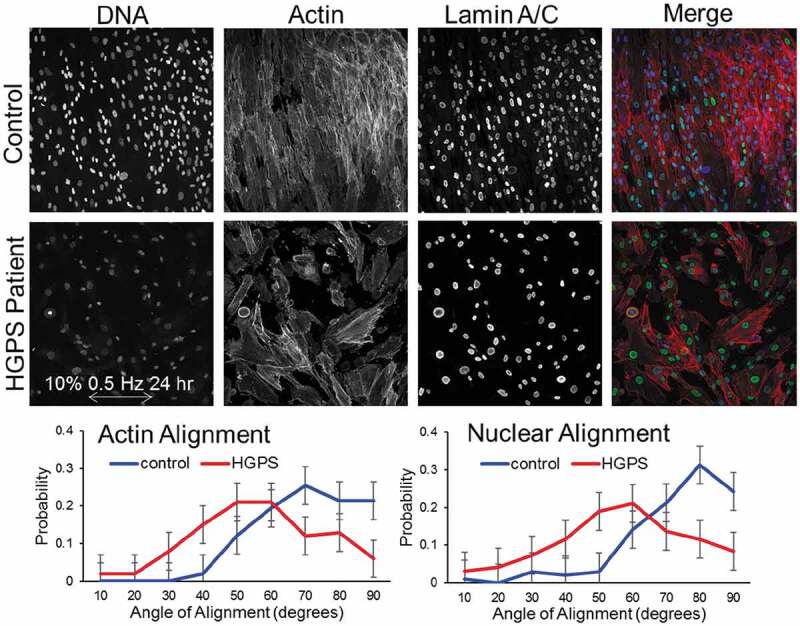
HGPS patient fibroblasts do not align under uniaxial stretch. Fibroblasts from a patient with HGPS or parent control were grown on deformable substrates and stretched at 0.5 Hz for 10% for 24 hr. Control cells showed characteristic orthogonal alignment to the applied stress with both actin and nuclei reorienting perpendicular to the direction of the stress. HGPS patient cells showed no particular alignment patterns. Comparatively, cell and nuclear shapes were also heterogeneous and irregular. Quantification (n > 100 cells per condition) shows alignment of actin fibers and nuclei preferentially 90° from the direction of stress. HGPS cells show mostly random distribution with some preference for 40°-70° distributions, but these are skewed by drastic cell shape differences. Error bars reflect sample size by Fisher’s exact method.

In addition to defects associated with exogenous progerin-expressing cells, we also aimed to examine cells from HGPS patients. HGPS patient fibroblasts are available from the Coriell Institute, along with HGPS control fibroblasts. Fibroblasts have previously been shown to align when the substrate is deformed perpendicular to the applied stretch [26]. The alignment of nuclei and actin cytoskeletal structures align dependent, to some degree, on frequency of stretch, amount of stretch and integrated time of stretch [27]. Control primary fibroblasts showed actin and nuclear alignment, but HGPS patient cells did not (Figure 5). Despite similar, high initial seeding densities between control and HGPS patient cells, there was substantial cell loss in the HGPS patient cells under stretch, likely due to cell death or detachment possibly due to the inability to adapt under force. Given the nearly complete lack of alignment as well as the heterogeneous shape of the HGPS cells (Figure 5) it was difficult to quantify the lack of alignment in the HGPS sample.

## Discussion

Nuclei in cells from patients with HGPS can exhibit protrusion of the nucleus toward the cytoplasm [11] as well as many other gross nuclear morphological changes [8,14]. There are many structural changes associated with HGPS including reduced lamin B1 levels [28], loss of heterochromatin [12], changes in chromatin-lamin binding [29], altered lamin-nuclear envelope association [30], altered nuclear pore complex [31] and changes in how the nucleus binds to the cytoskeleton [25]. Here, we have tried to examine lamina-specific defects through different cellular manipulations of cells exogenously expressing progerin. Of note by our group and others is that the exogenous expression of progerin, by plasmid such as DsRed-progerin or virus such as HA-progerin, is not the same as HGPS. Defects that result from exogenous expression appear to be more severe from the higher expression levels [7]. However, the physical model we propose here is entirely consistent with the force-induced wrinkling behavior observed in nuclei from patients with HGPS [8]. In previous studies, micropipette aspiration of isolated nuclei from patients shows wrinkling under high stress that is independent of the direction of applied force [8]. Thus, it appears that this model would hold with endogenous expression as well as with exogenous expression.

Several other lamin and nuclear envelope mutations are associated with nuclear dysmorphisms [32], and the term ‘blebbing’ has been used to categorize most of these altered shapes [33]. Although progerin-induced lamin misalignment may be due to altered signaling, it might not be mutually exclusive. We suggest here that the unique aspects of the nuclear shape changes – outward blebbing seen in some nuclear defects [34] versus the folds observed in HGPS – are likely significant markers of the etiology of this mechanical dysfunction. We suggest that the phrase blebbing should be used exclusively for an increase in the size of the nuclear envelope and an outward distention of a particular region of the nucleus. Thus, models developed for other nuclear blebs as outward protrusions and dilations may not necessarily be applied to progeria [3,35,36]. However, ‘traditional’ outward nuclear blebs have been observed in nuclei from progeria patients [11], which may be a function of passage time and other cellular factors suggesting numerous lamina failure mechanisms.

### Micro-aggregate model of the HGPS nuclear lamina

The energy of bending for an elastic two-dimensional surface that bends into a third dimension can be calculated based on previous works by Israelachvili [37]. Lamina networks are mostly elastic [38–40], and weak bending is a type of deformation that costs significantly less energy than stretching. The bending modulus, κ, of a general single elastic sheet is defined according to:

$$\kappa =\frac{1}{12}K_{stretch}h^{2}$$

where *h* is thickness and is the *K_stretch_* dilation modulus [37]. For the lamina of progerin-expressing cells, the *K_stretch_* would increase [8] and the local thickness, *h*, of the lamina increases significantly with progerin accumulation, as has been shown by electron and light micrographs [8,11]. Thus, κ would be much higher for progerin-expressing cells over control cells. Micropipette aspiration has confirmed the increased stiffening of the lamina nuclei from cells exogenously expressing progerin [7] in addition to nuclei from patients with HGPS measured by micropipette [8] and by stretching [9].

The resulting energy, *e_bend_*, to bend around a segment radius of curvature, *R*, can be described as [37]:

$$e_{bend}=\frac{1}{2}\kappa \frac{1}{R^{2}}$$

Since nuclei in both control and progerin-expressing cells show wrinkles and invaginations (Figure 3), in many cases with progerin-expressing cells showing more wrinkles then we assume that *R* doesn’t change or gets larger. Thus, if the energy required to bend the progerin lamina was much higher than a control lamina, then the deflections should be much smaller than control lamina. However, this is not the case. Another mechanism governing the wrinkling of the progerin lamina must be occurring.

**Figure 6.**
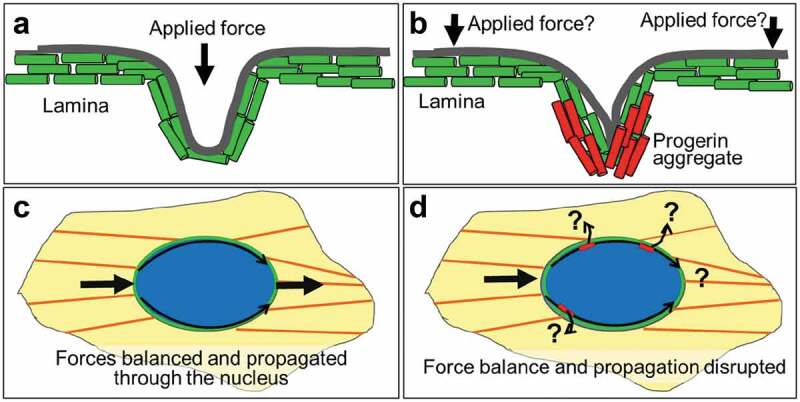
Model of nuclear lamina under force. (a) The nuclear lamina for control cells experiences a thinning of membrane and dilation of lamin A network. (b) The nuclear lamina for progerin-expressing cells experience high stress and buckle at the aggregates irrespective of force application. Wrinkles then emanate from the aggregate space. (c) In control cells cytoskeletal forces are balanced through the nuclear lamina and are propagated from one side of the nucleus to the other. (d) With wrinkles or defects in progerin-expressing cells forces may be disrupted along the lamina.

Given the differential responsiveness on patterns (Figure 3) and this model prediction, we suggest in sum that the deformations in the lamina of progeria cells are driven by entirely or mostly different factors that those seen in control cells. In Figure 6, we summarize a model that conveys the mechanism that we suggest for the nuclear lamina wrinkles associated for progerin. For control cells, due to a uniform distribution of lamin, stress, and exogenous forces cause the nuclear lamina to become thinner due to the elastic properties of the lamina [40] and therefore results in a dilation of the lamina network and low intensity values at the site of applied force (Fig. 56). Conversely, progerin-expressing cells show microaccumulations of progerin and deformation occurs at these regions rather than at regions of applied force (Figure 6(b)). This model will always show high intensities of progerin associated with defects. Figure 1 shows increased progerin intensity at the invaginations and Figure 5 shows defects growing from regions of high intensity. Also, this model accounts for defects that occur in regions not necessarily associated spatially with the application of force (Figures 2 and 3, wrinkles not aligned with actin filaments), rather defects associate with the region of accumulation of progerin. This model is consistent with our simulations as well as the concepts of stiffened inclusions shown in many examples throughout materials science.

Previous models of blebs have suggested that the lamina is restorative and resistant to blebs [35,36]. Finite element analysis of an isotropic elastic two-dimensional sheet has predicted folds rather than blebs in shape bifurcation studies, but not at regions distinct from applied pressure [41]. Also, the nature of intermediate filaments makes the lamina resistant to holes and defects from loss of local filament structure [42]. However, as with the HGPS defects seen here, there may be unrepairable damage to the lamina associated with overaccumulation.

### Implications in force transmission through the lamina and nucleus

One particularly important implication for the progerin-expressing nucleus would be structural integration of the cytoskeleton with the nucleoskeleton called the LINC (linker of nucleus to cytoskeleton) complex. The LINC complex is important in balancing forces throughout the cell and transmitting forces across the cell (Figure 6(c))[43,44]. Severing the LINC complex prevents forces from being transmitted to the inside of the nucleus [45] and forces from being transmitted from one side of the cell to the other [46]. It is unclear if LINC components are changed in HGPS cells. However, even if LINC complexes are maintained, we suggest that improper distribution of forces across the nuclear lamina from the non-isotropic distribution of lamins associated with progerin expression could modify the propagation of force throughout the cell (Figure 6(d)). This may be in-part why the wrinkles form away from the direction of the actin filaments in progerin-expressing cells (Figures 3 and 4). Thus, in HGPS premature aging, and in aspects of normal cellular aging [47], accumulated nuclear lamina defects may prevent proper force transmission through cells.

## Conclusions

Our findings reveal that the abnormal nuclear morphology observed in HGPS and progerin expression is a consequence of both structure and mechanics. Excessive accumulation of progerin at the nuclear lamina causes wrinkles and invaginations observed in numerous cellular conditions. We suggest that these altered shapes are a result of microaggregates rather than just a uniform stiffening of the lamina.

## Supplementary Material

Supplemental Material

## References

[1] Shimi T, Kittisopikul M, Tran J, et al. Structural organization of nuclear lamins A, C, B1, and B2 revealed by superresolution microscopy. Mol Biol Cell. 2015;26(22):4075–4086. 26310440 10.1091/mbc.E15-07-0461 PMC4710238

[2] Turgay Y, Eibauer M, Goldman AE, et al. The molecular architecture of lamins in somatic cells. Nature. 2017;543(7644):261–264. 28241138 10.1038/nature21382 PMC5616216

[3] Nmezi B, Xu J, Fu R, et al. Concentric organization of A- and B-type lamins predicts their distinct roles in the spatial organization and stability of the nuclear lamina. Proc Natl Acad Sci U S A. 2019;116(10):4307–4315. 30765529 10.1073/pnas.1810070116 PMC6410836

[4] Xie W, Chojnowski A, Boudier T, et al. A-type lamins form distinct filamentous networks with differential nuclear pore complex associations. Curr Biol. 2016;26(19):2651–2658. 27641764 10.1016/j.cub.2016.07.049

[5] De Sandre-Giovannoli A, Bernard R, Cau P, Navarro C, Amiel J, Boccaccio I, Lyonnet S, Stewart CL, Munnich A, Le Merrer M, Lévy N. Lamin a truncation in Hutchinson-Gilford progeria. Science. 2003;300(5628):2055. 12702809 10.1126/science.1084125

[6] Scaffidi P, Misteli T. Good news in the nuclear envelope: loss of lamin A might be a gain. J Clin Invest. 2006;116(3):632–634. 16511598 10.1172/JCI27820 PMC1386112

[7] Booth EA, Spagnol ST, Alcoser TA, et al. Nuclear stiffening and chromatin softening with progerin expression leads to an attenuated nuclear response to force. Soft Matter. 2015;11(32):6412–6418. 26171741 10.1039/c5sm00521c

[8] Dahl KN, Scaffidi P, Islam MF, et al. Distinct structural and mechanical properties of the nuclear lamina in Hutchinson-Gilford progeria syndrome. Proc Natl Acad Sci U S A. 2006;103(27):10271–10276. 16801550 10.1073/pnas.0601058103 PMC1502447

[9] Verstraeten VL, Ji JY, Cummings KS, et al. Increased mechanosensitivity and nuclear stiffness in Hutchinson-Gilford progeria cells: effects of farnesyltransferase inhibitors. Aging Cell. 2008;7(3):383–393. 18331619 10.1111/j.1474-9726.2008.00382.x PMC2651412

[10] Dahl KN, Kalinowski A, Pekkan K. Mechanobiology and the microcirculation: cellular, nuclear and fluid mechanics. Microcirculation. 2010;17(3):179–191. 20374482 10.1111/j.1549-8719.2009.00016.x PMC2881226

[11] Goldman RD, Shumaker DK, Erdos MR, et al. Accumulation of mutant lamin A causes progressive changes in nuclear architecture in Hutchinson-Gilford progeria syndrome. Proc Natl Acad Sci U S A. 2004;101(24):8963–8968. 15184648 10.1073/pnas.0402943101 PMC428455

[12] Shumaker DK, Dechat T, Kohlmaier A, et al. Mutant nuclear lamin A leads to progressive alterations of epigenetic control in premature aging. Proc Natl Acad Sci U S A. 2006;103(23):8703–8708. 16738054 10.1073/pnas.0602569103 PMC1472659

[13] Ferri G, Storti B, Bizzarri R. Nucleocytoplasmic transport in cells with progerin-induced defective nuclear lamina. Biophys Chem. 2017;229:77–83. 28712764 10.1016/j.bpc.2017.06.003

[14] Choi S, Wang W, Ribeiro AJS, et al. Computational image analysis of nuclear morphology associated with various nuclear-specific aging disorders. Nucleus. 2011;2(6):570–579. 22127259 10.4161/nucl.2.6.17798 PMC3324345

[15] Scaffidi P, Misteli T. Reversal of the cellular phenotype in the premature aging disease Hutchinson-Gilford progeria syndrome. Nat Med. 2005;11(4):440–445. 15750600 10.1038/nm1204 PMC1351119

[16] Cao K, Capell BC, Erdos MR, et al. A lamin A protein isoform overexpressed in Hutchinson-Gilford progeria syndrome interferes with mitosis in progeria and normal cells. Proc Natl Acad Sci U S A. 2007;104(12):4949–4954. 17360355 10.1073/pnas.0611640104 PMC1821129

[17] Eisch V, Lu X, Gabriel D, Djabali K. Progerin impairs chromosome maintenance by depleting CENP-F from metaphase kinetochores in Hutchinson-Gilford progeria fibroblasts. Oncotarget. 2016;7(17):24700–24718. 27015553 10.18632/oncotarget.8267 PMC5029735

[18] Capell BC, Erdos MR, Madigan JP, et al. Inhibiting farnesylation of progerin prevents the characteristic nuclear blebbing of Hutchinson-Gilford progeria syndrome. Proc Natl Acad Sci U S A. 2005;102(36):12879–12884. 16129833 10.1073/pnas.0506001102 PMC1200293

[19] Datta S, Snow CJ, Paschal BM. A pathway linking oxidative stress and the Ran GTPase system in progeria. Mol Biol Cell. 2014;25(8):1202–1215. 24523287 10.1091/mbc.E13-07-0430 PMC3982987

[20] Arsenovic PT, Ramachandran I, Bathula K, et al. Nesprin-2G, a component of the nuclear LINC complex, is subject to myosin-dependent tension. Biophys J. 2016;110(1):34–43. 26745407 10.1016/j.bpj.2015.11.014 PMC4805861

[21] Vaziri A, Mofrad MRK. Mechanics and deformation of the nucleus in micropipette aspiration experiment. J Biomech. 2007;40(9):2053–2062. 17112531 10.1016/j.jbiomech.2006.09.023

[22] Qin Z, Kalinowski A, Dahl KN, et al. Structure and stability of the lamin A tail domain and HGPS mutant. J Struct Biol. 2011;175(3):425–433. 21635954 10.1016/j.jsb.2011.05.015 PMC3150306

[23] Kalinowski A, Qin Z, Coffey K, et al. Calcium causes a conformational change in lamin A tail domain that promotes farnesyl-mediated membrane association. Biophys J. 2013;104(10):2246–2253. 23708364 10.1016/j.bpj.2013.04.016 PMC3660631

[24] Versaevel M, Grevesse T, Gabriele S. Spatial coordination between cell and nuclear shape within micropatterned endothelial cells. Nat Commun. 2012;3:671. 22334074 10.1038/ncomms1668

[25] Hale CM, Shrestha AL, Khatau SB, et al. Dysfunctional connections between the nucleus and the actin and microtubule networks in laminopathic models. Biophys J. 2008;95(11):5462–5475. 18790843 10.1529/biophysj.108.139428 PMC2586579

[26] Steward RL Jr., Cheng C-M, Wang DL, et al. Probing cell structure responses through a shear and stretching mechanical stimulation technique. Cell Biochem Biophys. 2010;56(2–3):115–124. 20033625 10.1007/s12013-009-9075-2

[27] Lee CF, Haase C, Deguchi S, et al. Cyclic stretch-induced stress fiber dynamics - dependence on strain rate, Rho-kinase and MLCK. Biochem Biophys Res Commun. 2010;401(3):344–349. 20849825 10.1016/j.bbrc.2010.09.046

[28] Dreesen O, Ong PF, Chojnowski A, et al. The contrasting roles of lamin B1 in cellular aging and human disease. Nucleus. 2013;4(4):283–290. 23873483 10.4161/nucl.25808 PMC3810336

[29] Bruston F, Delbarre E, Östlund C, et al. Loss of a DNA binding site within the tail of prelamin A contributes to altered heterochromatin anchorage by progerin. FEBS Lett. 2010;584(14):2999–3004. 20580717 10.1016/j.febslet.2010.05.032 PMC2908524

[30] Chojnowski A, Ong PF, Wong ES, et al. Progerin reduces LAP2alpha-telomere association in Hutchinson-Gilford progeria. Elife. 2015;4. DOI:10.7554/eLife.07759. PMC4565980 26312502

[31] Kelley JB, Datta S, Snow CJ, et al. The defective nuclear lamina in Hutchinson-gilford progeria syndrome disrupts the nucleocytoplasmic Ran gradient and inhibits nuclear localization of Ubc9. Mol Cell Biol. 2011;31(16):3378–3395. 21670151 10.1128/MCB.05087-11 PMC3147792

[32] Burke B, Stewart CL. Functional architecture of the cell’s nucleus in development, aging, and disease. Curr Top Dev Biol. 2014;109:1–52. 24947235 10.1016/B978-0-12-397920-9.00006-8

[33] Lüke Y, Zaim H, Karakesisoglou I, Jaeger VM, Sellin L, Lu W, Schneider M, Neumann S, Beijer A, Munck M, Padmakumar VC, Gloy J, Walz G, Noegel AA. Nesprin-2 Giant (NUANCE) maintains nuclear envelope architecture and composition in skin. J Cell Sci. 2008;121(11):1887–1898. 18477613 10.1242/jcs.019075

[34] Bercht Pfleghaar K, Taimen P, Butin-Israeli V, et al. Gene-rich chromosomal regions are preferentially localized in the lamin B deficient nuclear blebs of atypical progeria cells. Nucleus. 2015;6(1):66–76. 25738644 10.1080/19491034.2015.1004256 PMC4615727

[35] Funkhouser CM, Sknepnek R, Shimi T, et al. Mechanical model of blebbing in nuclear lamin meshworks. Proc Natl Acad Sci U S A. 2013;110(9):3248–3253. 23401537 10.1073/pnas.1300215110 PMC3587257

[36] Wren NS, Zhong Z, Schwartz RS, et al. Modeling nuclear blebs in a nucleoskeleton of independent filament networks. Cell Mol Bioeng. 2012;5(1):73–81. 22523521 10.1007/s12195-011-0196-5 PMC3328866

[37] Israelachvili JN. Intermolecular and surface forces. 3rd ed., Vol. xxx, Burlington, MA: Academic Press; 2011. p. 674 p.

[38] Dahl KN, Engler AJ, Pajerowski JD, et al. Power-law rheology of isolated nuclei with deformation mapping of nuclear substructures. Biophys J. 2005;89(4):2855–2864. 16055543 10.1529/biophysj.105.062554 PMC1366783

[39] Dahl KN, Kahn SM, Wilson KL, Discher DE. The nuclear envelope lamina network has elasticity and a compressibility limit suggestive of a molecular shock absorber. J Cell Sci. 2004;117(Pt 20):4779–4786. 15331638 10.1242/jcs.01357

[40] Rowat AC, Lammerding J, Ipsen JH. Mechanical properties of the cell nucleus and the effect of emerin deficiency. Biophys J. 2006;91(12):4649–4664. 16997877 10.1529/biophysj.106.086454 PMC1779937

[41] Kim DH, Li B, Si F, et al. Volume regulation and shape bifurcation in the cell nucleus. J Cell Sci. 2015;128(18):3375–3385. 26243474 10.1242/jcs.166330 PMC4582398

[42] Qin Z, Buehler MJ. Flaw tolerance of nuclear intermediate filament lamina under extreme mechanical deformation. ACS Nano. 2011;5(4):3034–3042. 21384869 10.1021/nn200107u

[43] Kaminski A, Fedorchak GR, Lammerding J. The cellular mastermind(?)-mechanotransduction and the nucleus. Prog Mol Biol Transl Sci. 2014;126:157–203. 25081618 10.1016/B978-0-12-394624-9.00007-5 PMC4591053

[44] Neelam S, Dickinson RB, Lele TP. New approaches for understanding the nuclear force balance in living, adherent cells. Methods. 2016;94:27–32. 26115785 10.1016/j.ymeth.2015.06.014 PMC4690816

[45] Spagnol ST, Dahl KN. Active cytoskeletal force and chromatin condensation independently modulate intranuclear network fluctuations. Integr Biol (Camb). 2014;6(5):523–531. 24619297 10.1039/c3ib40226f

[46] Alam SG, Lovett D, Kim DI, et al. The nucleus is an intracellular propagator of tensile forces in NIH 3T3 fibroblasts. J Cell Sci. 2015;128(10):1901–1911. 25908852 10.1242/jcs.161703 PMC4457156

[47] Scaffidi P, Misteli T. Lamin A-dependent nuclear defects in human aging. Science. 2006;312(5776):1059–1063. 16645051 10.1126/science.1127168 PMC1855250

