# Supplementary material for: Lamin microaggregates lead to altered mechanotransmission in progerin-expressing cells
Source: Nucleus. 2020 Aug 20;11(1):194–204. doi: 10.1080/19491034.2020.1802906 (PMC7529416; doi:10.1080/19491034.2020.1802906)
Supplement: Supplemental Material [file KNCL_A_1802906_SM9315.docx]

**Supplemental Figures**

**
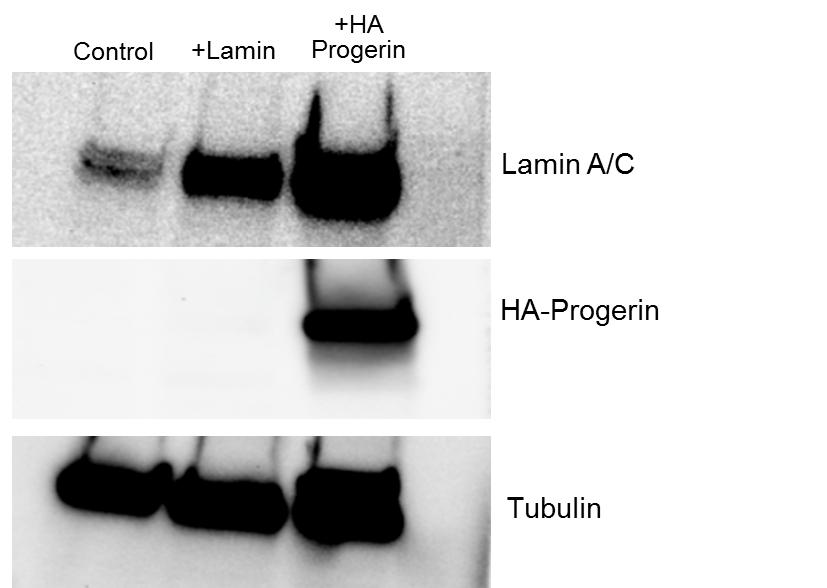
**

**Supplemental Figure 1:** Western blot of lamin A/C overexpression and HA-progerin expression in HUVEC to determine approximate protein levels from adenovirus.


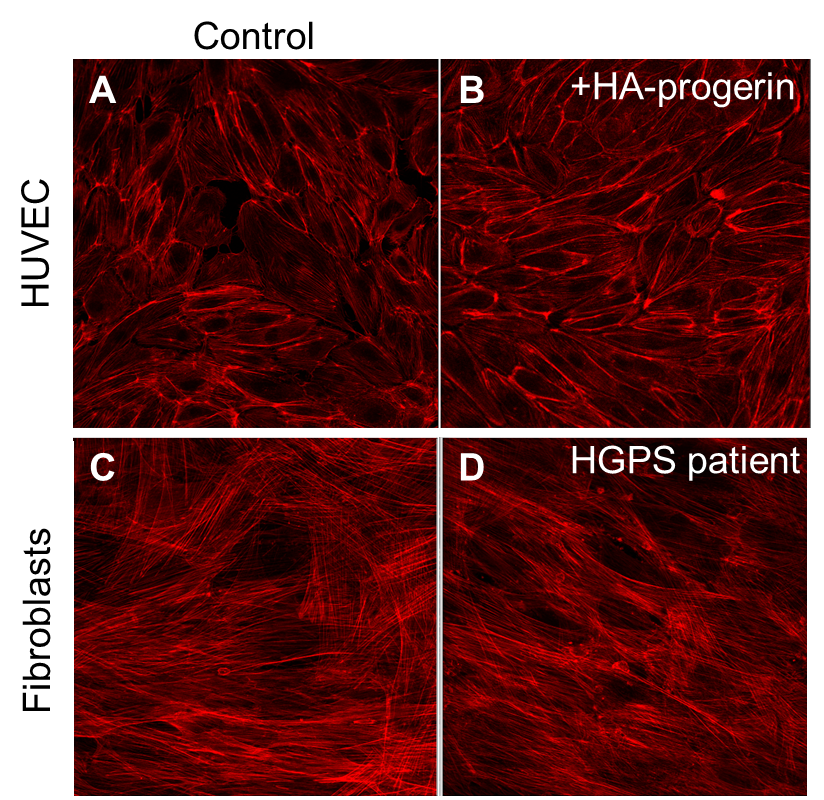


**Supplemental Figure 2: Comparison of actin fiber organization of HGPS patient cells and HA-Progerin model system.** A) Control HUVEC. B) HA-Progerin HUVEC. C) Control Fibroblasts. D) HGPS-Patient Fibroblasts. We did not observe any differences in actin organization with progerin expression compared to control cells. Furthermore, there were no observed differences between progerin expression in HUVEC to HGPS patient fibroblasts.


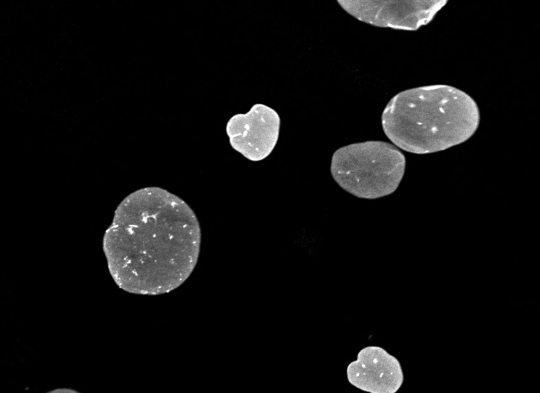


**Supplemental Figure 3: Punctate inclusions in progerin patient fibroblast nuclei.** Progeria patient fibroblasts stained with Lamin A/C and imaged with Zeiss confocal microscope. The same microaggregates are observed (arrows) in patient cells as they are seen in model system of HA-progerin.


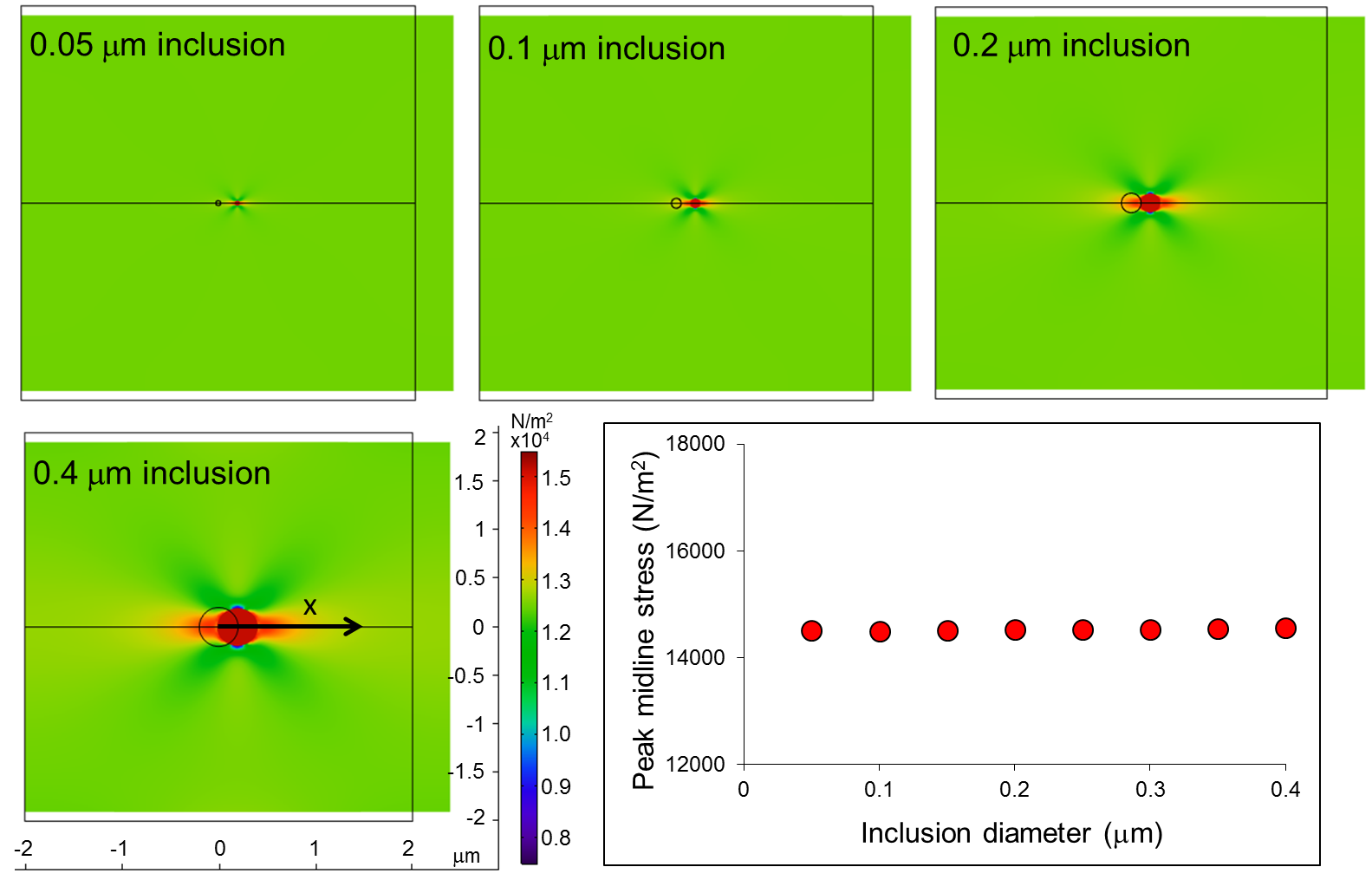


**Supplemental Figure 4: Simulations for measuring peak midline stress as a function of inclusion size.** Changes in size of an inclusion (1:2 inclusion:material stiffness) shown as 50, 100, 200 and 400 nm in a 4 μm by 4 μm square matching the stiffness of the nuclear lamina (50 kPa). The inclusion causes a midline stress along the x-axis, and the peak midline stress is independent of the inclusion size.


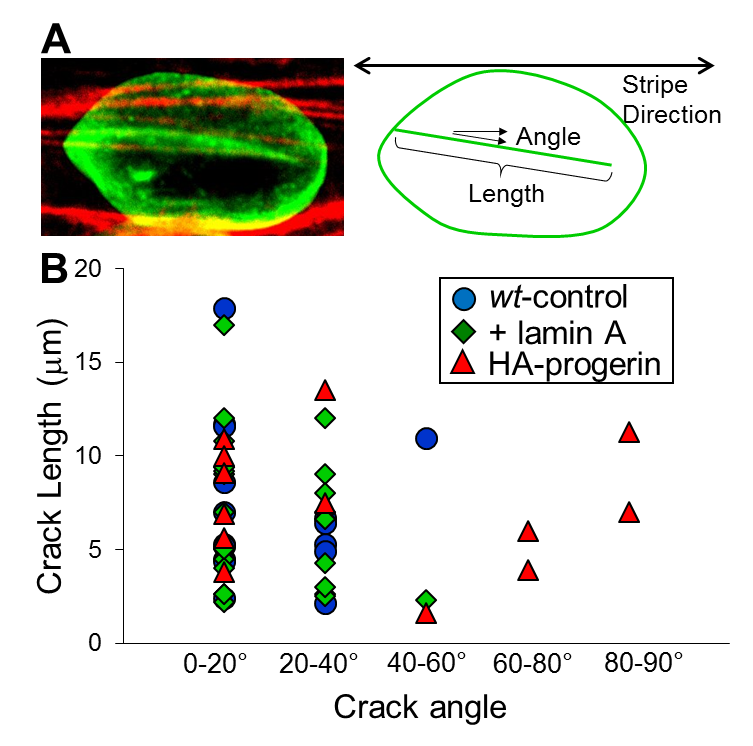


**Supplemental Figure 5: Methodology of measuring the angle and length of wrinkles.** A) We measure the length of the wrinkle or crack and the angle of the wrinkle with respect to the actin patterned stripe. B) Comparison of crack length versus crack angle shows no particular correlation.


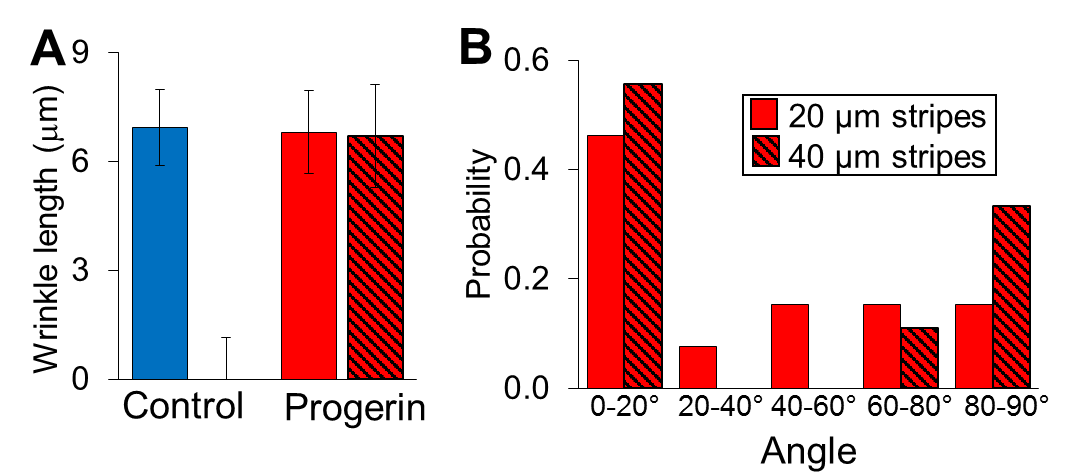


**Supplemental Figure 6: Wrinkle comparison on 40 μm stripes versus 20 μm stripes.** A) Length of deformations or wrinkles for control or HA-progerin expressing endothelial cells on 20 µm or 40 µm diameter stripes. On 40 µm stripes, control cells show no wrinkles whereas cells expressing progerin do. B) For progerin-expressing cells, orientation preference of the wrinkles is further lost as the stripe diameter widens. 30-50 cells per condition considered.
